# Comparison of in-brace correction between a (TLSO) using a floating pad with adjustable straps and a standard anterior open TLSO with no window openings for the treatment of congenital and infantile scoliosis: a case series

**DOI:** 10.1186/1748-7161-8-S2-O52

**Published:** 2013-09-18

**Authors:** David Speers, Katie Fisher

**Affiliations:** 1 Scheck and Siress Orthotics and Prosthetics, Chicago, IL, USA

## Background

Many styles of TLSOs can be used in the treatment of congenital and infantile scoliosis. A standard anterior open TLSO design with no window openings is one of the more common styles. A specific TLSO called the “Kalibus” was developed for the treatment of congenital scoliosis in very young children. The Kalibus consists of a rigid pelvic section, a floating pad with adjustable tension straps with a large opening opposite the pad and a rigid shoulder cuff. Typically, an inflexible spine characterizes congenital scoliosis, however, evaluating supine traction x-rays can determine spine flexibility as well as whether bracing is a treatment option to reduce some of the flexible deformities of the curve(s). 

## Purpose

This study compared the in-brace x-ray correction between a Kalibus-style TLSO and a standard anterior open design with no window openings. 

## Methods

For this study, three children (ages 2 years 8 months, 20 months and 11 months), two with a diagnosis of congenital scoliosis with some flexibility observed in traction x-rays and one with infantile scoliosis, were treated with both a Kalibus-style TLSO and a standard anterior open TLSO with no window openings. Initial in-brace x-rays were evaluated by one orthopedic surgeon. 

## Results

The standard anterior open TLSO provided corrections of 7 degrees and 8 degrees in the congenital scoliosis patients and 3 degrees in the infantile scoliosis patient. The Kalibus-style TLSO provided corrections of 0 degrees and 7 degrees in the congenital scoliosis patients and 0 degrees in the infantile scoliosis patient. 

## Conclusions and discussion

In this case series of three patients, the standard anterior open TLSO with no window openings produced greater in-brace correction than the Kalibus-style TLSO with a floating, adjustable pad with an opening opposite the pad. Additionally, we found that parents preferred the standard anterior open design as it was easier to manage than the Kalibus style. 
